# Selective regime shifts and intracellular gene transfer in the organellar genomes of the mycoheterotrophic orchid *Galeola nudifolia* Lour. (Orchidaceae)

**DOI:** 10.3389/fpls.2026.1923915

**Published:** 2026-09-09

**Authors:** Young-Kee Kim, Sangjin Jo, Rusea Go, Edward Entalai Besi, Christina Yong Seok Yien, Jin-Hyub Paik, Jun-Ho Song

**Affiliations:** 1 Advanced-Basic-Convergence Research Institute, Chungbuk National University, Cheongju, Republic of Korea; 2 International Biological Material Research Center, Korea Research Institute of Bioscience and Biotechnology, Daejeon, Republic of Korea; 3 Department of Biology, Universiti Putra Malaysia, Serdang, Malaysia; 4 Department of Biology, Chungbuk National University, Cheongju, Republic of Korea

**Keywords:** mitogenome, mycoheterotrophy, Orchidaceae, plastome, selection pressure, Vanilloideae

## Abstract

The transition to mycoheterotrophy has occurred repeatedly in Orchidaceae and is expected to impose contrasting evolutionary signatures on the plastome and mitogenome. However, quantitative comparisons between these two organellar compartments remain scarce, particularly within the Vanilloideae. Here, we report the first paired plastome and mitogenome of *Galeola nudifolia* Lour., a fully mycoheterotrophic orchid in the tribe Vanilleae (Vanilloideae), and integrate these data with published organellar genomes across photosynthetic and mycoheterotrophic Orchidaceae. The 852,490 bp mitogenome (24 linear contigs) encodes 38 protein-coding genes, eight tRNA genes, and three rRNA genes; the loss of *rpl*2 was confirmed by targeted re-assembly. The 101,856 bp plastome retained a typical quadripartite structure but exhibited extensive gene loss in photosynthesis-related genes. Forty-two plastome-derived regions, corresponding to 20 plastid protein-coding genes, were detected in the mitogenome; none retained intact open reading frames, indicating that physical transfer was extensive but functional transfer was absent. Branch-model (codeml) and RELAX analyses indicated a contrasting selective regime between the two genomes: relaxation of purifying selection in plastome genes was concentrated in the photosynthesis- and ATP synthesis-related loci (with *atp*I significant after multiple-testing correction, *p_adj_* = 0.033, and further ATP synthase genes supported by RELAX), whereas the mitogenome largely retained purifying selection. Predicted RNA-editing density showed no substantial differences between trophic groups, though this analysis is exploratory. In the plastome-based phylogeny, *Cyrtosia* was recovered as paraphyletic with respect to *G. nudifolia*; however, given the limited sampling and weak support, this arrangement is preliminary and requires confirmation with denser sampling and nuclear data.

## 1 Introduction

The transition to a non-photosynthetic lifestyle has occurred repeatedly and independently across terrestrial plants. Mycoheterotrophy has evolved at least 50 times in 17 families, and more than 30 of these origins have occurred within Orchidaceae Juss (Merckx and Freudenstein, 2010). Since the plastome, but not the mitogenome, encodes photosynthesis-related genes, the loss of photosynthesis is expected to leave asymmetric evolutionary signatures between the two organellar genomes. However, the nature and consistency of these signatures remain poorly understood across independent mycoheterotrophic origins.

Angiosperm plastomes are generally conserved in terms of gene content and structure. Since the first angiosperm plastome was reported (Shinozaki et al., 1986), 15,212 plastomes have been deposited in the National Center for Biotechnology Information (NCBI) database (accessed April 28, 2026). They encode approximately 79 protein-coding genes, four ribosomal RNAs (rRNAs), and 30 transfer RNAs (tRNAs). Their quadripartite structure comprises two single-copy regions, a large single-copy region (LSC) and a small single-copy region (SSC), separated by a pair of inverted repeats (IR-A and IR-B). The total length is typically 140–160 kb. This conservation breaks down in non-photosynthetic taxa. Photosynthesis-related genes are progressively lost, and the plastome is often reduced in size. Such reductions have been reported across distantly related heterotrophic lineages, including parasitic plants (e.g., Orobanchaceae Vent.), and mycoheterotrophs (e.g., Orchidaceae and Ericaceae Juss.) (Wicke et al., 2016; Graham et al., 2017). These are interpreted as a consequence of relaxed selective constraints on photosynthesis-related genes following the loss of photosynthetic capacity (Kim et al., 2023a). Therefore, the trophic shift directly shapes plastome evolution. In contrast, structural variation is less straightforward to interpret. Boundary shifts of IR/SC and other rearrangements have also been documented in photosynthetic taxa (Sinn et al., 2018; Rabah et al., 2019). Therefore, such structural changes are not unique to trophic transitions.

Angiosperm mitogenomes have been completely assembled far less often than plastomes, because they are difficult to assemble. They contain extensive repeats, undergo frequent recombination, and often incorporate promiscuous DNA from the plastid and nuclear genomes (Štorchová and Krüger, 2024). As a result, only 688 mitogenomes have been deposited in the NCBI database (accessed April 28, 2026), in stark contrast to the 15,212 plastomes available. In contrast, mitogenome gene content is generally conserved whereas size and structure are highly variable. Angiosperm mitogenomes typically encode 30–40 protein-coding genes, three rRNA genes, and 15–22 tRNA genes. Across terrestrial plants, reported mitogenome sizes span three orders of magnitude, from 66 kb in the epiphyte *Viscum scurruloideum* Barlow (Skippington et al., 2015) to 18.99 Mb in the gymnosperm *Cathaya argyrophylla* Chun & Kuang (Huang et al., 2025). Multichromosomal organization has been documented in photosynthetic taxa (e.g., *Dendrobium* Sw (Wang et al., 2023; Soe et al., 2025), *Silene noctiflora* L (Sloan et al., 2012)) and non-photosynthetic taxa (e.g., *Chamaegastrodia shikokiana* Makino & F. Maek (Kim et al., 2026), *Gastrodia* R.Br (Kim et al., 2023b; Wang et al., 2025), *Hypopitys monotropa* Crantz (Shtratnikova et al., 2020)). Structural rearrangements in plastomes occur preferentially at repeat-rich loci and tRNA gene positions (Haberle et al., 2008). Similarly, multichromosomal organization in mitogenomes occurs through recombination mediated by large repeats (Sloan, 2013). Repeat-associated recombination likely contributed to the structural evolution in both organellar genomes.

The evolutionary consequences of the loss of photosynthesis in organellar genomes have been primarily investigated in parasitic plants (Skippington et al., 2015; Cusimano and Wicke, 2016; Feng and Wicke, 2025). However, mycoheterotrophs fundamentally differ in their nutritional mechanisms. They acquire carbon indirectly through mycorrhizal fungal partners rather than through direct haustorial connections with the host plant (Merckx, 2012). Therefore, the loss of photosynthesis is expected to impose an asymmetric shift in the selective regime between the two organellar genomes. Photosynthesis-related genes encoded in the plastome are therefore expected to experience relaxed selective constraints, as their products are no longer functionally required. In contrast, respiratory genes encoded in the mitogenome remain essential for cellular respiration and ATP synthesis, regardless of how carbon is acquired. Therefore, they are expected to be maintained under purifying selection.

Among the lineages in which mycoheterotrophy evolved repeatedly, the Orchidaceae and the Ericaceae are prominent families. These are the only two families in which both organellar genomes have been characterized in mycoheterotrophic taxa. Plastomes have been reported in mycoheterotrophic orchids (Kim et al., 2017; Barrett et al., 2024; Liu et al., 2024) and angiosperms (Lam et al., 2018), while mitogenomes have been reported in mycoheterotrophic Ericaceae (Shtratnikova et al., 2020). Of these, the Orchidaceae is more tractable for testing the relationship between trophic mode and organellar genome evolution. The family contains approximately 28,000 species and is one of the two largest families of flowering plants (Chase et al., 2015; Christenhusz and Byng, 2016). It is also one of the few families in which the full gradient of mycoheterotrophy (i.e., initial, partial, and full mycoheterotrophy) is represented within a single phylogenetic framework (Merckx, 2012). Within the Orchidaceae, plastomes have been the primary focus of evolutionary studies (Givnish et al., 2015; Kim et al., 2020). In contrast, mitogenomic investigations have largely been confined to the genus *Gastrodia* (Yuan et al., 2018; Kim et al., 2023b; Wang et al., 2025). Plastome-only data are available for additional mycoheterotrophic orchids such as *Wullschlaegelia* Rchb.f (Barrett et al., 2024). Plant mitogenomes frequently incorporate plastid-derived sequences, a process that provides a record of intracellular gene transfer (IGT) between the two organellar compartments (Štorchová and Krüger, 2024). Testing such transfers, together with the joint evolution of organellar genomes, requires paired plastome and mitogenome data, which remain limited. As of April 28, 2026, such paired data were available for only 29 species across ten genera: *Apostasia* Blume (n = 2) (Ke et al., 2023), *Bletilla* Rchb.f. (n = 1) (Xiao et al., 2026), *Cyrtosia* Blume (n = 1) (Lee et al., 2025), *Chamaegastrodia* Makino & F. Maek. (n = 1) (Kim et al., 2026), *Cymbidium* Sw. (n = 3) (Li et al., 2023; Shen et al., 2024), *Dendrobium* (n = 6) (Wang et al., 2023; Soe et al., 2025), *Gastrodia* (n = 11) (Yuan et al., 2018; Kim et al., 2023b; Wang et al., 2025), *Hygrochilus* Pfitzer (n = 1), *Paphiopedilum* Pfitzer (n = 1) (Yang et al., 2023), and *Phalaenopsis* Blume (n = 2) (Chen et al., 2020). Of these ten genera, only three (*Cyrtosia*, *Chamaegastrodia*, and *Gastrodia*) are fully mycoheterotrophs. Therefore, the available data are too sparse to rigorously test hypotheses on organellar genome evolution during the loss of photosynthesis. In particular, trophic representation is insufficient.

Two gaps remain in our understanding of organellar genome evolution in mycoheterotrophy. First, asymmetric organellar genome evolution following the loss of photosynthesis has been demonstrated in some parasitic plants (Skippington et al., 2015). However, comparable quantitative tests for mycoheterotrophic lineages are scarce. Second, the plastomes of mycoheterotrophic lineages accumulate gene losses (Graham et al., 2017), IR/SC boundary shifts (Schelkunov et al., 2015), and accelerated substitution rates (Lam et al., 2015). These features can bias the plastome-based topologies, most notably through long-branch attraction (Lam et al., 2018). In contrast, angiosperm mitogenome coding regions generally evolve more slowly (Wolfe et al., 1987; Drouin et al., 2008). In addition, they are not subjected to mycoheterotrophy-related gene loss. Therefore, the mitogenome-based phylogenies offer a logically independent test for plastome-based topologies; however, such tests are rare. Both gaps are particularly evident in the Vanilloideae Szlach. Vanilloideae is one of five subfamilies of Orchidaceae. It is well suited for examining the link between trophic shifts and organellar genomic evolution. This subfamily includes both photosynthetic and fully mycoheterotrophic genera within a single phylogenetic framework (Chase et al., 2015). Photosynthetic representatives include *Vanilla* Plum. ex Mill. and *Pogonia* Juss. Fully mycoheterotrophic representatives include *Cyrtosia* and *Lecanorchis* Blume. Studies of organellar genomes in this subfamily have focused almost entirely on plastomes (Kim et al., 2019; Kim et al., 2023a; Zhou et al., 2023) with only a draft mitogenome reported for *Cyrtosia lindleyana* Hook.f. & Thomson. To date, this draft mitogenome has not been used to address the phylogenetic placement of mycoheterotrophic Vanilloideae, nor has it been used to evaluate the selective regimes of mitogenomes.

*Galeola* Lour. belongs to the tribe Vanilleae. This genus comprises 6–10 species (Iwatsuki et al., 1993; Chen and Cribb, 2007; Chase et al., 2015). Under the current taxonomy, five species are recognized: *Galeola faberi* Rolfe, *G. nudifolia* Lour., *G. cathcartii* Hook.f., *G. humblotii* Rchb.f., and *G. keo* Schuit. & M.Forbes (Borsch et al., 2020). All five species are fully mycoheterotrophic. They are distributed primarily throughout tropical Asia. However, *G. humblotii* extends to Madagascar and Comoros (Chen and Cribb, 2007). *Galeola* is closely related to *Cyrtosia* in molecular phylogeny (Givnish et al., 2016). The morphological boundary between the two genera is historically ambiguous, and species identification has therefore been difficult (Lee et al., 2025). As a fully mycoheterotrophic lineage of Vanilloideae, *Galeola* is a key taxon because of its phylogenetic relationships within Vanilloideae and the trajectory of organellar genome evolution following the loss of photosynthesis. Organellar genome data from this genus have only been reported for *Cyrtosia lindleyana* [≡ *Galeola lindleyana* (Hook.f. & Thomson) Rchb.f.] (Zhou et al., 2023; Lee et al., 2025). To address this gap, we sequenced and assembled the plastome and mitogenome of *Galeola nudifolia*, which is the most widely distributed of the five recognized species. This provides the first paired plastome and mitogenome dataset for *Galeola*. We integrated these data with those of the previously reported plastomes and mitogenomes of photosynthetic and mycoheterotrophic Orchidaceae within a unified phylogenetic framework.

This study addresses the following specific objectives: 1) to characterize the structural and genic features of the plastome and mitogenome of *G. nudifolia*, and to compare them with those of other Vanilloideae and other mycoheterotrophic orchids; 2) to reconstruct both plastome- and mitogenome-based phylogenies of Vanilloideae and to test whether the two organellar datasets yield congruent or conflicting phylogenetic placements of *G. nudifolia*; 3) to identify and characterize plastome-derived regions within the mitogenome, thereby assessing the extent of intracellular gene transfer between the two organellar compartments; and 4) to compare the selective regime between the plastome and the mitogenome of mycoheterotrophic Vanilloideae to characterize how the two organellar genomes differ following the loss of photosynthesis.

## 2 Materials and methods

### 2.1 Sampling, DNA extraction, and sequencing

Whole plants of *Galeola nudifolia*, including the root system, were obtained from Malaysia (Perak, Lenggong, Kampung Sumpitan). Species identification was based on morphological examination and confirmed by Professor Dr. Rusea Go (**Supplementary Figure 1**). A voucher specimen was deposited in the Herbarium of Universiti Putra Malaysia (UPM; accession number QA189). Since endophytic fungi predominantly colonize the root tissues and to minimize fungal contamination, silica-dried floral tissues and bracts were preferentially used for genomic DNA extraction. Tissues were homogenized into a fine powder in liquid nitrogen. Total genomic DNA was isolated using a DNeasy Plant Mini Kit (Qiagen, Hilden, Germany) following the manufacturer’s instructions. DNA quality and integrity were evaluated by agarose gel electrophoresis and the 4200 TapeStation System (Agilent Technologies, Santa Clara, CA, USA). The isolated genomic DNA was archived in the Chungbuk National University (CBU; accession number CBU-C-1360). Library preparation and 150-bp paired-end sequencing were performed by Macrogen (Seoul, Korea) using the Illumina NovaSeq 6000 platform (Illumina, San Diego, CA, USA), yielding approximately 100 Gb of raw reads. The initial quality assessment was conducted using FastQC v.0.11.9 (Andrews, 2010). Adapter sequences and low-quality bases were subsequently removed using Trimmomatic v.0.39 (Bolger et al., 2014), with the following parameters: ILLUMINACLIP: TruSeq3-PE-2.fa:2:30:10, LEADING:20, TRAILING:20, SLIDINGWINDOW:4:20, and MINLEN:36. The final dataset consisted of 737,340,014 trimmed reads.

### 2.2 Organellar genome assembly and annotation

The optimal k-mer size was determined using KmerGenie (Chikhi and Medvedev, 2013), which identified 37 as the optimal size. The trimmed reads were independently assembled in seed-based mode using two algorithmically distinct assemblers, GetOrganelle v.1.7.5 (Jin et al., 2020) and NOVOPlasty v.4.3.1 (Dierckxsens et al., 2017), to maximize the recovery of putative mitochondrial contigs. GetOrganelle was run with options -F embplant_mt, -R 20, -k 37, and -P 1000000, while NOVOPlasty, which requires a seed sequence, was run using protein-coding gene sequences of *Gastrodia elata* Blume as the seed (MF070084–MF070102), with a k-mer length of 37.

Putative mitochondrial contigs from both assemblers were identified by local BLASTN searches against a custom database of mitochondrial protein-coding genes derived from green plants (Kim et al., 2023b), and were then reconciled into a curated, non-redundant set. Contigs containing annotated protein-coding genes were prioritized; overlapping or redundant contigs recovered from the two assemblers were merged by assembly in Geneious v.10.2.6 (Kearse et al., 2012), with the resulting consensus sequences retained; and contigs were filtered by read-mapping coverage depth to remove low-coverage or spurious sequences. The retained contigs were verified by provisional annotation in Geneious against the complete mitogenome of *Gastrodia pubilabiata* Y.Sawa (GenBank OR004100–OR004143; Kim et al., 2023b). Although a mitogenome of the Vanilloideae species *Cyrtosia lindleyana* [≡ *Galeola lindleyana*] is publicly available, it was not fully or reliably annotated at the time of analysis. No completely and reliably annotated mitogenome was therefore available for Vanilloideae. For this reason, *G. pubilabiata* (Epidendroideae), for which a fully annotated mitogenome was publicly available, was selected as the reference for provisional annotation. This procedure yielded a curated set of first-draft mitochondrial contigs used as trusted input for subsequent hybrid assembly with SPAdes v.3.13.1 (Bankevich et al., 2012).

The provisional annotation indicated that *rpl*2, though present in *G*. *pubilabiata*, was missing from the first-draft contig set. To determine whether *rpl*2 was genuinely absent or had merely been missed during assembly, the *rpl*2 sequence from the *G*. *pubilabiata* mitogenome was used as a seed for an additional targeted NOVOPlasty assembly. This targeted assembly recovered no *rpl*2-containing contig despite adequate read coverage of the flanking regions, confirming that *rpl*2 is genuinely absent rather than missing due to an assembly gap.

Trimmed reads and pooled trusted contigs were subjected to error correction and hybrid assembly in SPAdes with k-mer sizes of 21, 37, 55, and 77. The resulting contigs were screened using local BLASTN searches against the same custom database of mitochondrial protein-coding genes (options: -evalue 0.01 and -outfmt 6). Verified mitochondrial contigs were manually curated within Geneious, using a built-in assembler. Trimmed reads were mapped to the assembled contigs using BWA v.0.7.19 (Li, 2013). The coverage depth was calculated using SAMtools v.1.21 (samtools depth) (Danecek et al., 2021). Given the prevalence of repetitive elements and structural rearrangements (which are characteristic of angiosperm mitogenomes), forced circularization was not pursued. Instead, 24 validated contigs were retained as linear sequences, constituting the draft mitogenome of *G. nudifolia*. Contigs were selected based on coverage depth and annotation content.

Final annotation of the mitochondrial contigs was performed using BLASTN (Altschul et al., 1990), tRNAscan-SE v.2.0 (Lowe and Chan, 2016), ‘ORFfinder,’ and the ‘Find Annotations’ function in Geneious. A total of 29 published orchid mitogenomes retrieved from NCBI (**Supplementary Table 1**) were used both as references for annotating the *G. nudifolia* mitogenome and, subsequently, were re-examined under the same criteria so that gene presence/absence could be assessed consistently across all taxa. Alternative start codons (ACG and TTG) were considered during open reading frame (ORF) prediction. Pseudogenes and gene loss were assessed according to criteria described previously (Kim et al., 2020). A linear map of the annotated mitogenome was generated using the OrganellarGenomeDRAW (OGDRAW) web server (Greiner et al., 2019).

In parallel, the plastome of *G. nudifolia* was assembled from the draft contigs generated by GetOrganelle and NOVOPlasty during the mitogenome assembly process. Putative plastome contigs were identified by filtering these draft contigs with BLASTN, and were then reassembled using the Geneious assembler to reconstruct the complete plastome sequence. To validate the assembly, the raw reads were mapped back to the reconstructed plastome using BWA and SAMtools, and the resulting coverage depth was examined to confirm assembly completeness and integrity. The plastome was annotated using BLASTN, tRNAscan-SE, and Geneious, and a circular map of the annotated plastome was generated using the OGDRAW web server.

### 2.3 Simple sequence repeats, dispersed repeats and RNA editing prediction

Simple sequence repeats (SSRs) in both the plastome and mitogenome were identified using Phobos v.3.3.12 (Mayer, 2010), with minimum repeat thresholds set at 10, 5, 3, 3, and 3 units for mono-, di-, tri-, tetra-, and pentanucleotide motifs, respectively. For these comparative repeat analyses, the plastome sequence of *Cyrtosia lindleyana* (NC064997) and mitogenome sequences of *C. lindleyana* (OQ716404–OQ716453), representing the only congeneric species with publicly available organellar genome data, were retrieved from the NCBI database. Dispersed repeats in both the plastome and mitogenome sequences of *G. nudifolia* and *C. lindleyana* were detected using the repeat finder tool in Geneious, with a minimum repeat length of 100 bp and no mismatches permitted. Repeat distribution, SSR composition, and syntenic relationships between *G. nudifolia* and *C. lindleyana* were visualized using Circos v.0.69 in TBtools-II (Krzywinski et al., 2009; Chen et al., 2023).

For each of the 30 orchid mitogenomes (**Supplementary Table 1**), including *Galeola nudifolia*, RNA editing sites were independently predicted using Deepred-Mt with default parameters (Edera et al., 2021). For each mitogenome, individual protein-coding gene sequences were extracted and used as input. Predicted sites with a posterior probability below 0.9 were excluded from further analysis. To compare the RNA editing patterns between trophic groups, the retained editing sites were compiled across all sampled taxa, which were classified as either mycoheterotrophic or photosynthetic based on their trophic mode. For each taxon, the number of predicted editing sites per gene was normalized by the coding sequence length, yielding the editing frequency expressed as sites per kilobase. Median editing frequencies were subsequently calculated per gene for each trophic group, and the difference in median editing frequencies between mycoheterotrophic and photosynthetic taxa was used to assess exploratory group-level variation in RNA editing patterns. Genes were classified into the following functional categories based on their annotated function: NADH dehydrogenase, ATP synthase, cytochrome complex, ribosomal proteins, maturase, and other. All statistical summaries and visualizations were performed using the *ggplot2* (Wickham et al., 2016), *ggrepel* (Slowikowski et al., 2024), and *patchwork* (Pedersen, 2019) packages in R.

### 2.4 Putative gene transfer survey

To investigate putative gene transfer events and their evolutionary origins, two data matrices established in previous studies were used as reference datasets based on plastomes and mitogenomes (Kim et al., 2023b), with taxonomic metadata incorporated to facilitate the accurate inference of evolutionary patterns (**Supplementary Table 1**). Local BLASTN searches were conducted to assess sequence homology and identify the putative origins of transferred regions (evalue ≤ 0.01). Overlapping BLASTN hit regions were consolidated using the merge function in BEDTools (Quinlan, 2014) and the resulting data were visualized using the *ggplot2* and *dplyr* packages in R (Wickham et al., 2016; R Core Team, 2021; Wickham et al., 2023).

Merged hit regions were examined to determine whether they encompassed protein-coding sequences. Protein-coding sequences were extracted from the reference data matrices and used to construct a dedicated BLAST database using makeblastdb. To distinguish functional from nonfunctional transfers, each plastome-derived region overlapping a plastid protein-coding gene was aligned to its corresponding full-length plastid gene extracted from the plastome, and its reading frame was inspected by translation. ORF integrity was assessed from alignment coverage and continuity together with the presence of internal stop codons: regions retaining a full-length, uninterrupted reading frame were considered potentially functional, whereas regions showing truncation, partial alignment, or internal stop codons were classified as nonfunctional (pseudogenized) transfers.

Given that fungal-derived sequences have been documented in orchid mitogenomes (Sinn and Barrett, 2020) and considering the mycoheterotrophic lifestyle of *G. nudifolia*, the proportion of fungal-derived sequences in the mitogenome was assessed as a comparative check for possible fungal contamination. Putative fungal-derived regions in the assembled mitogenome were identified by local BLASTN searches against the NCBI nucleotide (nt) database (e-value < 1e-5), counting any region with a significant hit to a fungal sequence. The same criterion was applied uniformly to all 30 orchid mitogenomes to allow relative comparison. Because significant hits were counted regardless of top-hit taxonomy, and conserved genes shared between plant and fungal genomes were not excluded, the absolute proportions may be overestimated; the metric was therefore used only as a relative, comparative proxy rather than as a definitive quantification of fungal-derived content or as evidence of horizontal gene transfer. On this basis, fungal-derived sequences accounted for 2.94% of the *G. nudifolia* mitogenome, within the range observed across the 29 orchid mitogenomes (0.6–3.4%; **Supplementary Table 2**). Comparable or higher proportions were found in several photosynthetic orchids, indicating that this fungal-derived content represents a general feature of orchid mitogenomes rather than contamination associated with the mycoheterotrophic lifestyle of *G. nudifolia*.

### 2.5 Phylogenetic analysis

Forty plastome sequences were retrieved from NCBI and combined with the newly assembled *G*. *nudifolia* plastome, resulting in 41 taxa for plastome-based phylogenetic analysis (**Supplementary Table 1**). Given the highly reduced plastomes characteristic of some mycoheterotrophic orchids, only the protein-coding and ribosomal RNA (rRNA) regions present across all sampled taxa were retained, yielding a subset of 23 protein-coding and four rRNA regions. Each region was individually aligned using MAFFT v.7.450 with default parameters (Katoh et al., 2002) and the resulting alignments were trimmed using trimAl v.1.5.rev0 following the default settings (Capella-Gutiérrez et al., 2009). The best-fit substitution model for each partition was determined using ModelFinder (Kalyaanamoorthy et al., 2017) as implemented in IQ-TREE2 v.2.3.6 (Minh et al., 2020), with the selected models summarized in **Supplementary Table 3**. Trimmed alignments were concatenated into a single supermatrix using AMAS (Borowiec, 2016), yielding a total alignment length of 37,007 bp. Maximum likelihood (ML) phylogenetic inference was conducted using IQ-TREE2 with partitioned models, and nodal support was evaluated using an ultrafast bootstrap approximation (1,000 replicates) (Hoang et al., 2017). Apostasioideae, which is sister to all other Orchidaceae and represents the earliest-diverging subfamily (Chase et al., 2015; Givnish et al., 2015), was used as the outgroup. The resulting phylogeny was visualized using TreeGraph v.2.15.0-887 (Stöver and Müller, 2010).

For mitogenome-based phylogenetic analysis, 29 orchid mitogenome sequences were retrieved from the NCBI (**Supplementary Table 1**). Together with the mitogenome of *G. nudifolia* generated in this study, a total of 30 mitogenome sequences were included in the phylogenetic analysis. Regions universally present across all sampled taxa were retained, comprising 38 protein-coding regions and three rRNA regions. Sequence alignment, trimming, substitution model selection, supermatrix construction, and ML inference were performed using MAFFT, trimAl, ModelFinder, AMAS, and IQ-TREE2, respectively, following the procedure described above for the plastome dataset, yielding a concatenated alignment of 44,628 bp. The concatenated alignment was also used for Bayesian phylogenetic inference. Bayesian inference (BI) was performed using MrBayes v3.2 under the GTR+I+Γ substitution model (Ronquist et al., 2012). The Markov chain Monte Carlo (MCMC) analysis was run for 20,000,000 generations, with trees and parameters sampled every 1,000 generations. The initial 2,000,000 generations were discarded as burn-in, and a majority-rule consensus tree was constructed from the remaining samples. Representatives of the subfamily Apostasioideae were designated as the outgroup, and the resulting phylogeny was visualized using TreeGraph.

### 2.6 Evolutionary analysis

To investigate the evolutionary rate variation across Orchidaceae, the plastome- and mitogenome-based analyses were restricted to taxa for which both organellar genomes were available, so that selective regimes could be directly compared between the two compartments. Mitogenome sequences were available for 30 taxa, six of which lacked a corresponding plastome; accordingly, 24 plastome and 30 mitogenome sequences were analyzed. This differs from the phylogenetic analyses, which used all available plastomes, because only plastome data are required for phylogenetic inference. A total of 74 protein-coding regions were examined in the plastome dataset. Because gene content varies extensively among mycoheterotrophic plastomes, each gene was analyzed using the subset of taxa in which it was retained, rather than requiring presence across all taxa; genes retained in no mycoheterotrophic tip could not be tested and were excluded. For the mitogenome dataset, 31 protein-coding regions were included, after excluding genes with extensive missing data across the sampled taxa. Each region was extracted and aligned using the codon-aware aligner MACSE v.2.07, following default parameters (Ranwez et al., 2018). Sequences harboring premature stop codons, which cause runtime errors in the downstream analyses, were excluded prior to rate estimation.

Variation in the nonsynonymous-to-synonymous substitution rate ratio (*ω* = d*N*/d*S*) was assessed on a gene-by-gene basis using the branch model implemented in PAML v.4.9 (codeml) (Yang, 2007). Two nested models were compared for each gene: a null model (H_0_) assuming a uniform ω across all branches, and an alternative model (H_1_) allowing distinct ω values for foreground and background branches. Mycoheterotrophic taxa were designated as the foreground branches, with photosynthetic species serving as the background branches.

The mitogenome-based phylogeny was used as a guide tree for both the plastome and mitogenome datasets. Although the two topologies were not fully congruent, the plastome-based phylogeny exhibited pronounced long branches in the mycoheterotrophic lineages, likely reflecting accelerated substitution rates in functionally relaxed plastid genes rather than true phylogenetic signal. In contrast, mitochondrial genes are retained across both photosynthetic and mycoheterotrophic taxa and are expected to provide a less biased representation of organismal relationships. Therefore, mitogenome-based phylogeny was considered more appropriate as a guide tree for branch-model analyses, in which mycoheterotrophic lineages constitute the foreground branches. Nonetheless, fixing a single guide tree for both datasets, particularly one whose topology is not fully resolved, may influence branch-length estimation, and this represents a limitation of the present analysis.

The model fit was evaluated using a likelihood ratio test (LRT), with twice the log-likelihood difference assessed against a chi-squared distribution with one degree of freedom. Raw *p*-values were corrected for multiple testing across all tested genes using the Benjamini–Hochberg false discovery rate (FDR) procedure (Benjamini and Hochberg, 1995). For downstream interpretation and visualization, genes were subsequently excluded based on two criteria: (i) optimization failure, defined as cases where the log-likelihood of H_1_ did not exceed that of H_0_ (LRT statistic ≤ 0), and (ii) boundary convergence, defined as ω estimates ≤ 0.001 or ≥ 10. The difference in *ω* between foreground and background branches was used as a quantitative measure of evolutionary rate shift in mycoheterotrophic lineages relative to photosynthetic taxa. We note that the plastome and mitogenome datasets differ in taxon sampling (24 vs. 30 taxa, respectively), which may affect cross-compartment comparisons of *ω* to an extent that is difficult to quantify without a fully overlapping taxon set. All results were visualized using the *ggplot2* (Wickham et al., 2016), *ggrepel* (Slowikowski et al., 2024), and patchwork (Pedersen, 2019) packages in R.

To formally distinguish relaxed from intensified selection—which cannot be inferred from elevated *ω* alone—we complemented the branch-model analysis with the RELAX 4.5 implemented in HyPhy 2.5.64 (Wertheim et al., 2015; Kosakovsky Pond et al., 2020). RELAX tests whether the intensity of selection, summarized by the parameter *K*, differs between a test and a reference set of branches, with *K* < 1 indicating relaxed and *K* > 1 intensified selection relative to the reference. Using the same alignments, gene set, and lineage assignment as the branch-model analysis, mycoheterotrophic lineages were designated as the test branches and photosynthetic lineages as the reference branches, and RELAX was run separately for each gene. Genes for which no mycoheterotrophic tip retained the locus (e.g., *ndh*B) could not be tested and were excluded. Because RELAX evaluates the direction and significance of the shift in selection intensity rather than a point estimate of its magnitude, we interpreted *K* in terms of its direction (relaxation vs. intensification) and statistical support rather than treating extreme *K* values as precise effect sizes. Raw *p*-values were corrected for multiple testing across all tested genes using the Benjamini–Hochberg procedure, independently of the branch-model analysis (**Supplementary Table 10**).

## 3 Results

### 3.1 Organellar genome characterization

**Figure 1:**
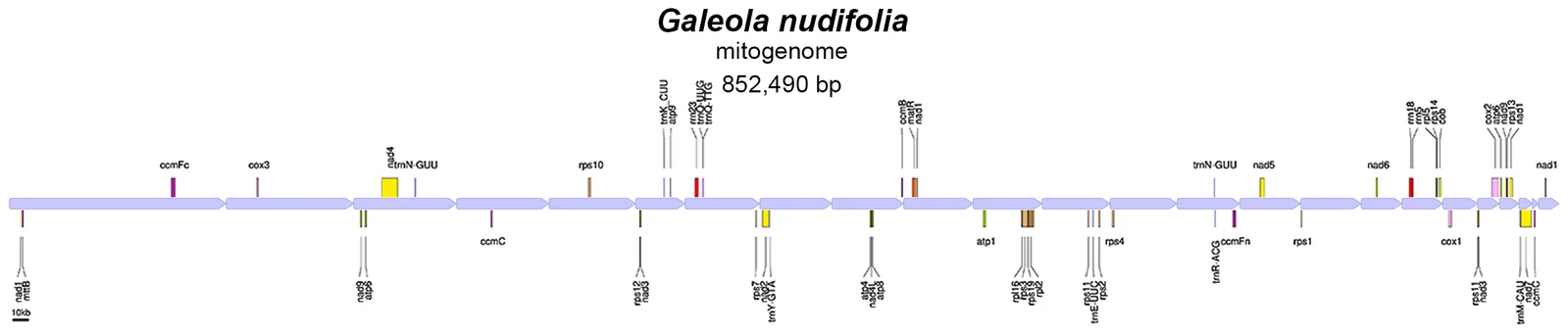
The mitogenome of *Galeola nudifolia* is composed of 24 mitochondrial contigs, with a total length of 852,490 bp. It includes 40 protein-coding genes, eight transfer RNA genes, and three ribosomal RNA genes.

**Figure 2:**
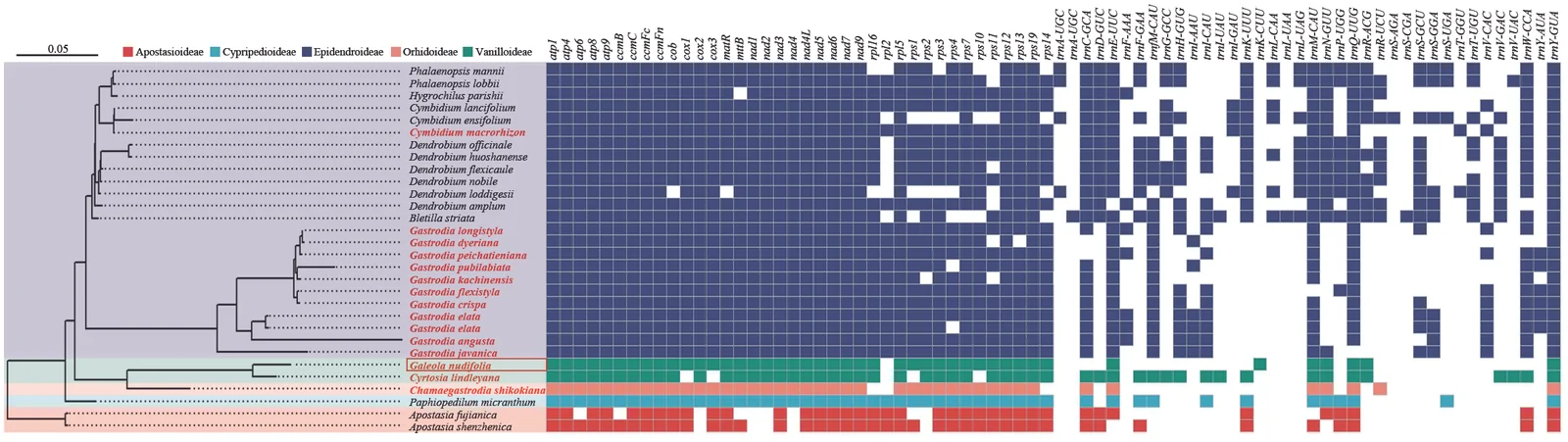
Heatmap of mitochondrial gene content across Orchidaceae. Colored boxes indicate gene presence, and white boxes indicate gene absence. The x-axis represents protein-coding genes and tRNA genes, while the y-axis represents the scientific names of orchid species. Subfamilies are distinguished by distinct color coding along the y-axis. Species names highlighted in red indicate mycoheterotrophic taxa. Gene presence/absence was assessed consistently across all taxa by re-examining each mitogenome under the same criteria (see Materials and Methods), rather than relying on the original GenBank annotations. Because raw sequencing reads were not available for the publicly sourced taxa, however, gene absences in these taxa could not be validated to the same extent as for the newly generated *G. nudifolia* mitogenome, and some apparent absences may reflect differences in assembly completeness, annotation criteria, or organellar read sorting rather than genuine gene loss.

The draft mitogenome of *Galeola nudifolia* was assembled into 24 contigs with a total length of 852,490 bp (**Table 1**; **Figure 1**). The overall GC content was 46.7%, with an average coverage depth of 651.98-fold. Owing to the prevalence of repetitive elements, structural rearrangements, and frequent recombination, the reconstruction of a complete circular mitogenome was not feasible. Consequently, the mitogenome was retained in a linear contig format. Thirty-eight protein-coding genes, eight tRNA genes, and three rRNA genes were annotated. Comparative analysis revealed the absence of *rpl*2 in *G. nudifolia*. Among the 29 other orchid mitogenomes compared, *rpl*2 was absent in 11 species and present in the remaining 18 (**Figure 2**). The absence in *G. nudifolia* was confirmed by targeted re-assembly (see Materials and Methods).

**Table 1:** General features of the mitogenome and the plastome of *Galeola nudifolia* and *Cyrtosia lindleyana*.

Scientific name	Region	Numbers	Length	GC	CDS	tRNAs	rRNAs	Coverage-depth
*Galeola nudifolia*	Plastome	1	101856	33.7	37 (4)	23 (3)	4	7627.66x
*G. nudifolia*	Mitogenome	24	852490	46.7	38	8	3	651.98x
*C. lindelyana*	Plastome	1	100749	34.4	32 (4)	20 (3)	4	–
*C.lindelyana*	Mitogenome	50	694243	47.5	34	9	2	–

Gene counts refer to unique genes; numbers in parentheses indicate genes duplicated in the inverted repeat (IR).

The plastome of *G*. *nudifolia* was 101,856 bp long, with an overall GC content of 33.7% and an average coverage depth of 7,627.66-fold (**Supplementary Figure 2**). The plastome exhibited the typical quadripartite structure characteristic of most angiosperms, consisting of an LSC region (61,059 bp) and an SSC region (17,709 bp) separated by two IR regions (11,544 bp each). A total of 64 unique genes were annotated, comprising 37 protein-coding genes, 23 tRNA genes, and four rRNA genes (**Table 2**). Of these, four protein-coding genes and three tRNA genes were duplicated in IRs.

**Table 2:** Genes contained in the *Galeola nudifolia* plastome.

Category	Group	Name of genes		
		Present	Pseudogenized	Absent
Self replication	rRNA genes	*rrn*4.5, *rrn*5, *rrn*16, *rrn*23		
	tRNA genes	23 tRNAs (3 tRNAs in IR)		
	Small subunit of ribosome	*rps*2, *rps*3, *rps*4, *rps*7, *rps*8, *rps*11, *rps*12*, *rps*14, *rps*15, *rps*16*, *rps*18, *rps*19 (x2)		
	Large subunit of ribosome	*rpl*2* (x2), *rpl*14, *rpl*16**, *rpl*20, *rpl*22, *rpl*23 (x2), *rpl*32, *rpl*33, *rpl*36		
	DNA dependent RNA polymerase		*rpo*A	*rpo*B, *rpo*C1, *rpo*C2
Photosynthesis-related	Subunits of NADH-dehydrogenase			*ndh*A, *ndh*B, *ndh*C, *ndh*D, *ndh*E, *ndh*F, *ndh*G, *ndh*H, *ndh*I, *ndh*J, *ndh*K
	Subunits of photosystem I		*psa*A, *psa*I	*psa*B, *psa*C, *psa*J
	Subunits of photosystem II	*psb*N, *psb*T, *psb*Z	*psb*A, *psb*B, *psb*C, *psb*H, *psb*M	*psb*D, *psb*E, *psb*F, *psb*I, *psb*J, *psb*K, *psb*L
	Subunits of cytochrome b/f complex	*pet*L		*pet*A, *pet*B, *pet*D, *pet*G, *pet*N
	Subunits of ATP synthase	*atp*A, *atp*B, *atp*E, *atp*F*, *atp*H, *atp*I		
	Large subunit of rubisco		*rbc*L	
Other genes	Translational initiation factor	*inf*A		
	Maturase	*mat*K		
	Protease	*clp*P**		
	Envelope membrane protein			*cem*A
	Subunit of Acetyl-CoA-carboxylase	*acc*D		
	c-type cytochrome synthesis gene			*ccs*A
	*ycf*	*ycf*1, *ycf*2 (x2)	*ycf*3	*ycf*4

One and two asterisks after gene names indicate genes containing one and two introns, respectively. Genes located in the inverted repeat (IR) regions are indicated by (x2). Additionally, the functional status of typically conserved plastid genes is distinguished as present, pseudogenized, or absent.

A total of 541 predicted RNA editing sites were retained in the mitogenome of *G. nudifolia* under a posterior probability cutoff of ≥ 0.9. All the retained editing sites were located in the *nad* genes (*nad*1, *nad*2, *nad*3, *nad*4, *nad*4L, *nad*5, *nad*6, *nad*7, and *nad*9; **Supplementary Table 4**). Editing sites in other mitochondrial genes were also predicted at lower probabilities but were excluded by the cutoff. To assess whether the predicted RNA-editing density differed between mycoheterotrophic and photosynthetic orchids, the predicted editing sites were compared across published orchid mitogenomes (**Supplementary Table 4**). No substantial differences in RNA-editing density were observed between the two trophic groups.

In total, 148 SSRs were identified within the mitogenome of *G*. *nudifolia*, including 14 mononucleotides, 27 dinucleotides, 29 trinucleotides, 57 tetranucleotides, and 21 pentanucleotide motifs (**Supplementary Table 5**). In comparison, the mitogenome of *C*. *lindleyana* contained 119 SSRs, including nine mononucleotide, 16 dinucleotide, 22 trinucleotide, 52 tetranucleotide, and 20 pentanucleotide motifs (**Supplementary Table 5**). In the plastome, *G. nudifolia* harbored 67 SSRs, comprising 27 mononucleotide, 20 dinucleotide, seven trinucleotide, eight tetranucleotide, and five pentanucleotide motifs whereas the plastome of *C. lindleyana* contained 71 SSRs (29 mononucleotide, 22 dinucleotide, four trinucleotide, 12 tetranucleotide, and four pentanucleotide motifs; **Supplementary Table 5**).

**Figure 3:**
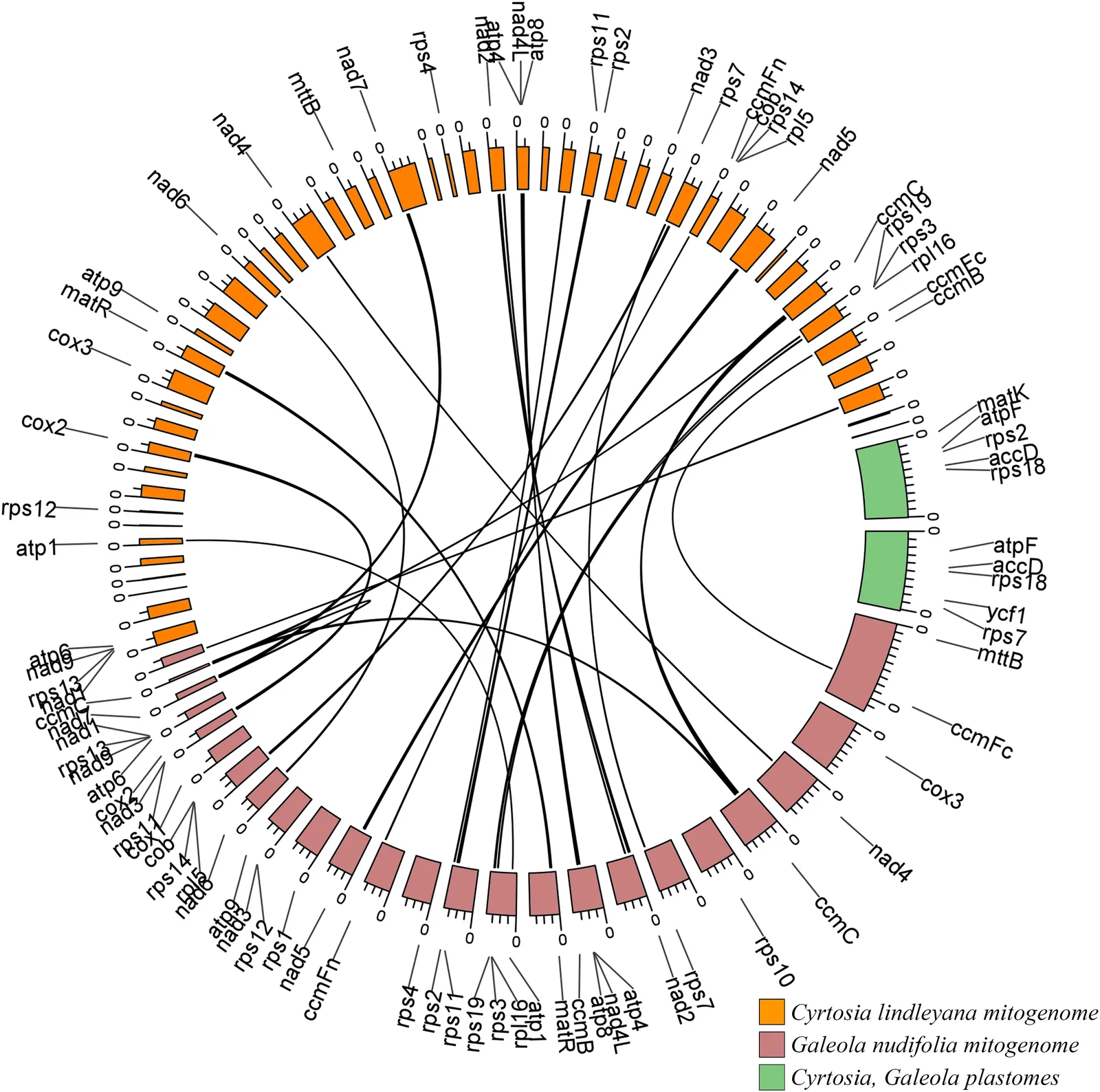
Comparative analysis of dispersed repeats in the plastome and mitogenome of *Cyrtosia lindleyana* and *Galeola nudifolia*. A total of 36 dispersed repeat regions were detected across the four organellar genomes, of which 34 were specific to mitogenomes and two were specific to plastomes; none were shared between the plastome and mitogenome of either species. Black lines connect homologous repeat regions within the mitogenomes. Scale ticks are placed at every 50 kb.

To investigate the dispersed repeats across organellar genomes, a comparative repeat analysis was performed for the plastome and mitogenome of *G*. *nudifolia* and *C*. *lindleyana*. Across the four organellar genomes (plastome and mitogenome of each species), a total of 36 dispersed repeats were detected. Of these, 34 were specific to mitogenomes, two were specific to plastomes, and none were shared between the two organellar compartments (**Figure 3**; **Supplementary Table 6**).

To identify putative plastome-derived sequences within the mitogenome of *G*. *nudifolia*, independent BLASTN searches were conducted against both the plastome and mitogenome reference databases. In total, 55,983 plastome-derived BLASTN hits were identified. These hits were merged into nonoverlapping regions using BEDTools, yielding 46 candidate regions for putative plastome-derived insertions (**Supplementary Tables 7**, **8**). Among these candidate regions, 42 demonstrated a higher hit count against the plastome database than the mitogenome database, suggesting potential plastome-derived insertions in the mitogenome. The remaining four regions showed equal or higher hit counts against the mitogenome database and were therefore excluded from further consideration as plastome-derived sequences. Based on BLASTN results, these 42 regions showed sequence similarity to 20 unique plastome protein-coding genes in the reference plastome dataset: *atp*A, *atp*B, *atp*F, *ndh*B, *ndh*C, *pet*B, *psb*B, *psb*C, *psb*D, *psb*H, *psb*T, *rbc*L, *rpl*16, *rpl*23, *rpo*C2, *rps*2, *rps*7, *ycf*1, *ycf*2, and *ycf*3. Although plastome-derived regions were widespread in the mitogenome, all coding regions were either truncated or contained internal stop codons and thus lacked intact reading frames, indicating that these transfers are pseudogenized and nonfunctional rather than functional gene relocations.

### 3.2 Phylogenetic relationships

**Figure 4:**
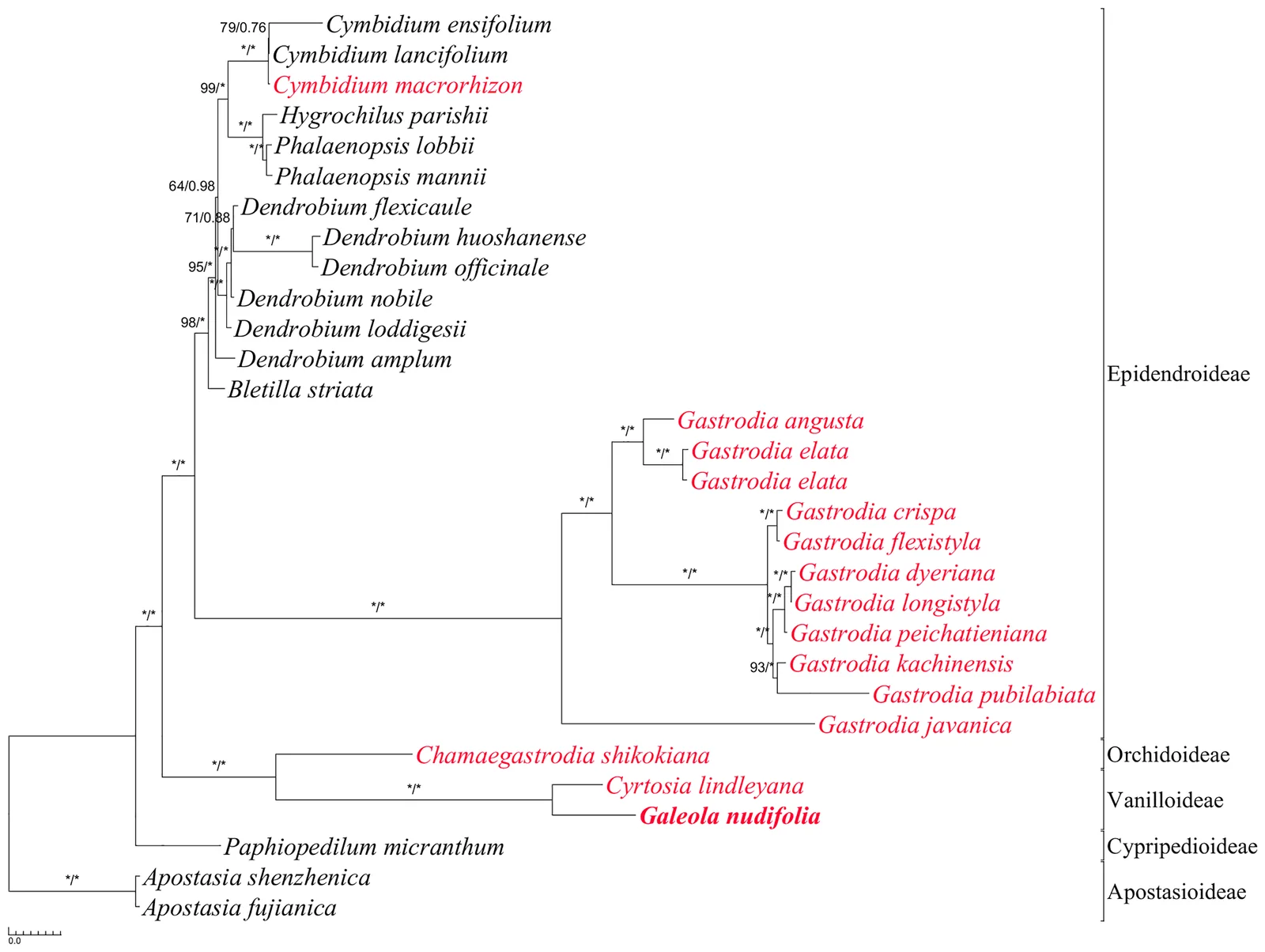
Maximum-likelihood phylogenetic tree of 30 orchid species based on concatenated nucleotide alignments of 38 mitochondrial protein-coding genes and three rRNA genes. The final alignment comprised 44,628 bp. Maximum-likelihood inference was performed using IQ-TREE 2 with the best-fit substitution models listed in **Supplementary Table 3**. Bayesian inference recovered an identical topology, obtained by MrBayes. Node support values are presented as maximum-likelihood bootstrap support/Bayesian posterior probability (ML/BI), with asterisks (*) representing values of 100% and 1.0, respectively. *Galeola nudifolia* is highlighted in bold, and nonphotosynthetic mycoheterotrophic species are shown in red.

In the mitogenome-based ML and Bayesian phylogenetic tree (**Figure 4**), *G. nudifolia* formed a clade with *C. lindleyana*. This Vanilloideae clade was recovered as sister to *Chamaegastrodia shikokiana* (Orchidoideae). The two analyses recovered identical topologies, differing only in the level of nodal support at three weakly supported nodes; support is reported below as bootstrap support (BS) and Bayesian posterior probability (PP). The mycoheterotrophic genera *Chamaegastrodia, Cyrtosia, Galeola*, and *Gastrodia* exhibited long branch lengths. Most nodes in the mitogenome-based phylogeny were strongly supported (BS ≥ 93%), with correspondingly high posterior probabilities, including the backbone relationships among subfamilies and the placement of the mycoheterotrophic Vanilloideae. A few nodes, however, received lower support: the node uniting Vandeae Lindl., Cymbidieae Pfitzer, and Malaxideae Lindl. (BS = 64%, PP = 0.98), the grouping of *Dendrobium flexicaule* Z.H.Tsi, S.C.Sun & L.G.Xu with (*D. huoshanense* Z.Z.Tang & S.J.Cheng, *D. officinale* Kimura & Migo) (BS = 71%, PP = 0.88), and the sister relationship of *Cymbidium ensifolium* (L.) Sw. and *C. lancifolium* Hook. (BS = 79%, PP = 0.76). These weakly supported nodes involve relationships that are not central to the placement of *G. nudifolia.*

In the ML tree based on plastome protein-coding genes, for which broader taxon sampling was available, *Cyrtosia nana* (Rolfe ex Downie) Garay and *G. nudifolia* formed a strongly supported clade (BS = 100%; **Supplementary Figure 3**). *C. lindleyana* was recovered as sister to this clade, although with low support (BS = 49%), and *C. septentrionalis* (Rchb.f.) Garay was placed successively outside them (BS = 100%). Under this topology, the three sampled *Cyrtosia* species do not form a monophyletic group but instead subtend *G. nudifolia* in a grade, rendering *Cyrtosia* paraphyletic with respect to *Galeola*. However, because the node uniting *C. lindleyana* with the (*C. nana*, *G. nudifolia*) clade is weakly supported (BS = 49%), and because sampling is limited to three *Cyrtosia* and one *Galeola* accession without nuclear data, this arrangement should be regarded as preliminary. This *Cyrtosia*–*Galeola* group was in turn recovered as sister to *Lecanorchis* (Vanilloideae; BS = 71%).

### 3.3 Substitution rate variation and selection pressure in organelles

To assess whether the transition to mycoheterotrophy altered selective pressures on organellar genes, branch model analyses were performed using codeml, with mycoheterotrophic lineages designated as the foreground branches. A total of 31 mitochondrial and 74 plastome protein-coding genes were analyzed (**Supplementary Table 9**).

**Figure 5:**
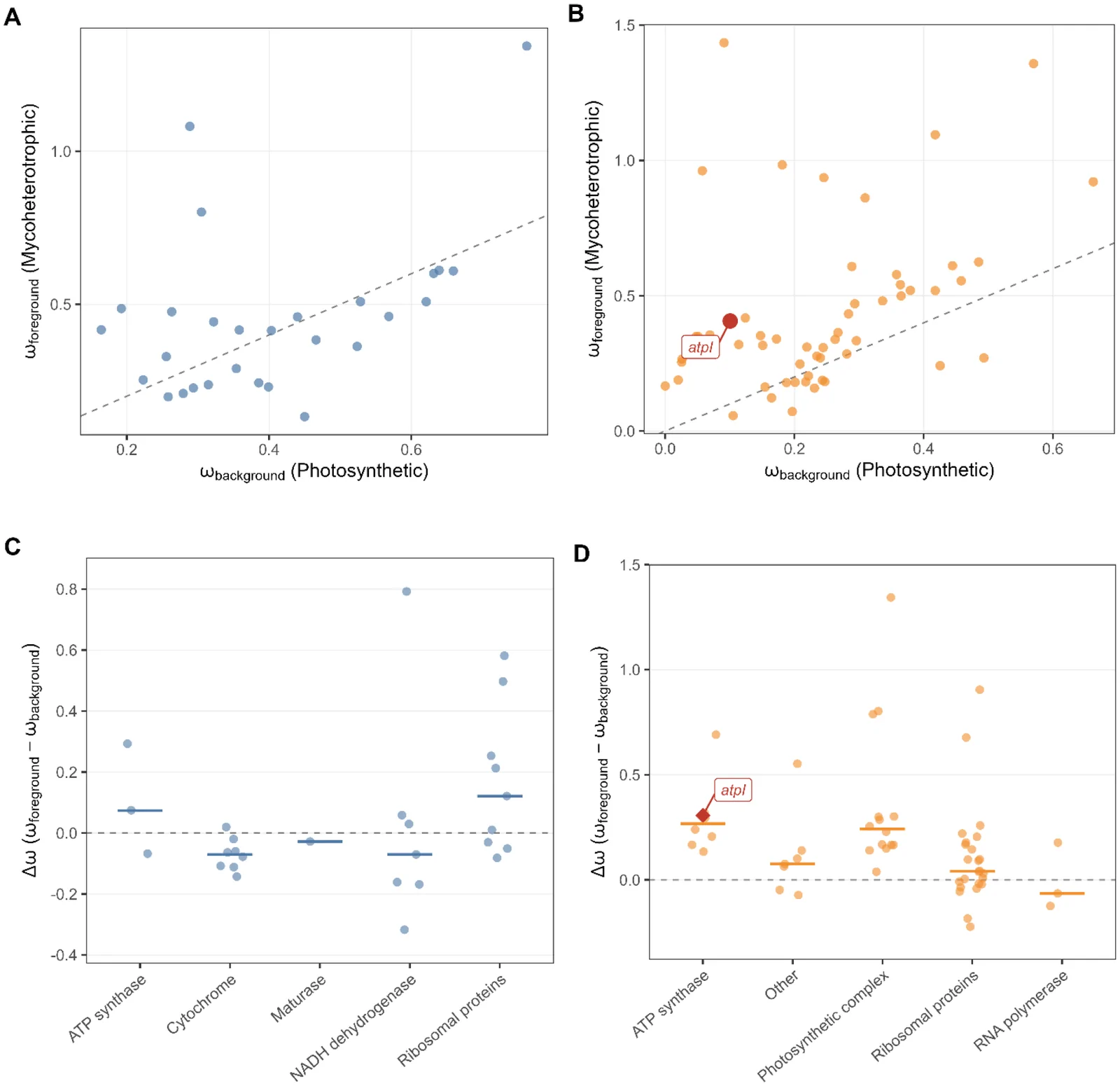
Comparison of selective pressures on mitochondrial and plastid protein-coding genes between mycoheterotrophic and photosynthetic orchids. **(A, B)** Scatter plots of the *dN/dS* ratio (ω) estimated for the background (photosynthetic orchids; x-axis) versus the foreground (mycoheterotrophic orchids; y-axis) lineages under the branch model implemented in PAML codeml, shown for the mitogenome **(A)** and plastome **(B)**. The diagonal line (*ω*_foreground_ = *ω*_background_) demarcates genes experiencing relaxed purifying selection (above the line) from those under enhanced purifying selection (below the line) in mycoheterotrophic orchids. **(C, D)** Dot plots of the difference in ω values (Δ*ω* = *ω*_foreground_ − *ω*_background_) for each protein-coding gene, grouped by functional category, shown for the mitogenome **(C)** and plastome **(D)**. Positive Δ*ω* values indicate relaxed purifying selection, and negative values indicate enhanced purifying selection in mycoheterotrophic orchids relative to photosynthetic orchids. The plastid gene *atp*I was the only gene with a statistically significant shift in selective pressure after Benjamini–Hochberg correction (*p_adj_* < 0.05), exhibiting pronounced relaxation of purifying selection. Note that the axis ranges differ between the mitogenome and plastome panels, reflecting the overall distribution of ω values in each genome compartment.

In the mitogenome, *ω* values in the foreground branches tended to be slightly elevated relative to the background, but the gene-wise rate shifts were modest (**Figure 5A**). No mitochondrial gene exhibited a statistically significant difference in *ω* between mycoheterotrophic and photosynthetic lineages after multiple testing correction (all *p_adj_* > 0.05; **Supplementary Table 9**). The background *ω* estimate for *rps*14 reached the upper boundary, likely reflecting the absence of synonymous substitutions in the background branches.

In contrast, plastome genes displayed a pronounced shift toward relaxed purifying selection in the mycoheterotrophic lineages (**Figure 5B**). Among the plastome genes tested, *atp*I was the only gene with a statistically significant elevation of ω in the foreground branches (*ω*_foreground_ = 0.41 vs. *ω*_background_ = 0.10; *p_adj_* = 0.033). Four additional genes showed elevated ω in the foreground branches with *p*_adj_ values just above the significance threshold (*atp*B, *atp*E, *atp*H, and *psb*E; all *p_adj_* = 0.054). These genes encode the subunits of photosynthetic complexes (*psb*E) and ATP synthase (*atp*B, *atp*E, and *atp*H). For three plastome genes (*cem*A, *rpl*23, and *rpo*A), the foreground *ω* estimates reached the upper boundary.

When Δ*ω* (*ω*_foreground_ − *ω*_background_) was compared across functional categories, mitogenome Δ*ω* values remained close to zero in all categories, with no discernible category-specific pattern (**Figure 5C**). Plastome genes associated with photosynthetic complexes and ATP synthase exhibited generally positive Δ*ω* values, indicative of relaxed purifying selection in mycoheterotrophic lineages (**Figure 5D**). However, plastome ribosomal protein and RNA polymerase genes showed Δ*ω* values near zero. Notably, mitogenome ATP synthase genes showed Δ*ω* near zero, whereas plastome ATP synthase genes showed elevated Δ*ω* comparable to plastome photosynthetic complex genes (**Figures 5C, D**).

To distinguish relaxed from intensified selection, we applied the RELAX framework to each organellar gene, with mycoheterotrophic lineages as the test set and photosynthetic lineages as the reference set (**Supplementary Table 10**). After FDR correction, 11 genes showed a significant shift in selection intensity. The majority were plastid genes undergoing relaxation (*K* < 1), and these were concentrated in the ATP synthase complex (*atp*A, *atp*B, *atp*E, *atp*H, and *atp*I), together with *psb*E and *mat*K. A smaller number of genes showed intensification (*K* > 1), including the plastid genes *ycf*2 and *rps*7. In the mitogenome, only two genes were significant: *nad*6, which showed relaxation, and *atp*4, which showed intensification. Full RELAX results, including *K* values and adjusted *p*-values for all genes, are provided in **Supplementary Table 10**.

## 4 Discussion

### 4.1 Mitogenome structural complexity and functional conservation

Orchid mitogenomes show extreme structural heterogeneity, making it difficult to identify general structural trends (Kim et al., 2026). Among the orchid mitogenomes reported to date, only two *Apostasia* mitogenomes (*A. fujianica* and *A. shenzhenica*) have a single circular structure (Ke et al., 2023). Even between these two congeneric species, the mitogenomes differ substantially, with at least six large inversion events. If such extensive rearrangements occur between congeneric species that share a single circular form, the more complex multichromosomal architecture observed in other orchid mitogenomes would not be surprising.

Within mycoheterotrophic species, the number of mitochondrial contigs varies widely, ranging from 16 to 73 in *Gastrodia* and 24–50 in Vanilloideae (**Supplementary Table 1**) (Yuan et al., 2018; Kim et al., 2023b; Lee et al., 2025; Wang et al., 2025). Photosynthetic orchids show comparable variation, indicating that the contig number does not track with trophic mode (Wang et al., 2023). The total mitogenome size showed the same pattern: mycoheterotrophic species spanned more than a five-fold range (412,787–2,246,035 bp), whereas photosynthetic orchids fell within a narrower range (479,649–746,435 bp; **Supplementary Table 1**). This broader size variation likely reflects species-specific factors such as repeat expansion, rather than the trophic mode itself.

The lack of an association between trophic mode and mitogenome structure extends to gene content. The protein-coding gene complement of the *G. nudifolia* mitogenome was comparable to that of the photosynthetic orchids (**Figure 2**). For *C. lindleyana*, the publicly available mitogenome (GenBank: OQ716404-OQ716453) was originally deposited with 17 annotated protein-coding genes; because the raw reads were not available, we re-annotated the deposited assembly by homology-based BLASTN searches against published orchid mitogenomes, recovering 34 protein-coding genes (**Figure 2**). The only protein-coding gene loss shared between the two Vanilloideae mitogenomes is *rpl*2. This loss is not specific to mycoheterotrophic lineages; independent *rpl*2 losses have been documented in 10 additional orchid species spanning both photosynthetic and mycoheterotrophic lineages across multiple subfamilies (**Figure 2**).

In contrast to the conservation of protein-coding genes, the mitochondrial tRNA composition showed greater variation in fully mycoheterotrophic species. Four tRNA genes (*trn*T-UGU, *trn*K-UUU, *trn*R-ACG, and *trn*V-GAC) are usually present in the mitogenomes of photosynthetic orchids, with occasional absences in some species and more frequent absences in fully mycoheterotrophic species (**Figure 2**). Plastome-derived tRNAs have been documented in the mitogenomes of *Chamaegastrodia* and *Gastrodia* (Kim et al., 2023b; Kim et al., 2026). Many mitochondrial tRNAs are of plastid origin, and differences in organellar read sorting during assembly can inflate or deflate tRNA inventories. In the present study, plastid-derived contamination was minimized using BLAST-based contig selection and coverage depth filtering. Nonetheless, differential tRNA distribution may reflect intracellular sequence transfer between organellar genomes, as tRNA-mediated recombination has been identified as a pivotal driver of plastome-to-mitogenome gene transfer (Haberle et al., 2008).

Overall, the protein-coding gene complement of the orchid mitogenome was not substantially altered by the transition to mycoheterotrophy, despite considerable structural variation and some differences in tRNA composition. This conservation of the protein-coding repertoire is consistent with the expectation that respiratory function and the mitochondrial genes encoding it remain essential regardless of the trophic mode.

### 4.2 Plastome organization and trophic modes

Plastome evolution in *Galeola* is characterized by distinct reductive trends and structural rearrangements. The plastome of *G. nudifolia* (101,856 bp; **Supplementary Figure 2**) is the largest among the closely related mycoheterotrophic Vanilleae genera, followed by *Cyrtosia* (85–100 kb) and *Lecanorchis* (70–74 kb). This progressive size reduction along the mycoheterotrophic gradient within Vanilleae, from the relatively intact *Galeola* and *Cyrtosia* plastomes to the severely reduced *Lecanorchis* plastomes, supports the established association between trophic mode and plastome degradation (Wicke et al., 2016; Graham et al., 2017).

However, unlike size reduction, structural rearrangements of the plastome, particularly IR boundary shifts and SSC relocation, appear to occur independently of the trophic mode. In typical orchid plastomes, the SSC region does not contain rRNA genes. However, in several mycoheterotrophic Vanilleae species (*Cyrtosia*, *Galeola*, and *Lecanorchis*), the SSC gene contents have been relocated, and the IR is contracted to contain only *ycf*2 (Kim et al., 2019; Kim et al., 2020; Kim et al., 2023a; Lee et al., 2025). Although these rearrangements might superficially appear to be consequences of mycoheterotrophy, several lines of evidence argue against this interpretation. First, photosynthetic Vanilleae species (*Pogonia* and *Vanilla*) exhibit extensively enlarged IR regions and shortened SSC regions (Kim et al., 2023a), demonstrating that similar rearrangements occur without changes in the trophic mode. Second, the mycoheterotrophic *Chamaegastrodia shikokiana* (Orchidoideae) possesses a *Vanilla*-like plastome structure with an enlarged IR and shortened SSC, rather than the pattern observed in its fellow mycoheterotrophs in Vanilleae (Kim et al., 2020). Third, the same IR expansion phenomenon was recently reported in two photosynthetic Epidendroideae genera, *Caularthron* Raf. and *Myrmecophila* Rolfe (Li et al., 2024). Collectively, these observations indicate that IR contraction and expansion are lineage-specific rather than trophic mode-specific. In addition to these structural rearrangements, the plastome of *G. nudifolia* has lost several genes that are typically associated with photosynthetic functions (**Table 2**; **Supplementary Figure 2**). The physical transfer of plastome-derived sequences into the mitogenome is extensive in *G. nudifolia*, raising the question of whether remnants of these lost genes persist in the mitogenome.

### 4.3 Plastome-derived insertions in the mitogenome

Intracellular gene transfer (IGT) from the plastome to the mitogenome is a widespread evolutionary phenomenon in land plants (Wang et al., 2007). Mitochondrial**-**targeted plastid transfers (MTPTs) are copy-based transfers in which the plastome retains its original sequence. In *G. nudifolia*, 42 putative plastome-derived regions were identified in the mitogenome. Of these, 23 regions—corresponding to 13 unique genes—are homologous to photosynthesis-related genes (*atp*, *ndh*, *pet*, *psb*, and *rpo* gene families; **Supplementary Table 8**). The plastome counterparts of most of these genes—*ndh*B, *ndh*C, *pet*B, *psb*B, *psb*C, *psb*D, *psb*H, *rbc*L, and *rpo*C2—are absent from the current plastome of *G. nudifolia* (**Supplementary Figure 2**), implying that these transfers likely predated the severe degradation of the plastome. In contrast, four genes still present in the plastome (*atp*A, *atp*B, *atp*F, and *psb*T) are also represented among the transferred regions, consistent with more recent or ongoing transfer. Together, this indicates that the plastome-derived content of the mitogenome accumulated through transfer events of differing ages. A comparable pattern has been reported in other mycoheterotrophic orchids: the mitogenomes of *Gastrodia* and *Epipogium* Borkh. retain *psb*A-homologous regions despite the absence of *psb*A from their plastomes (Kim et al., 2023b; Zhao et al., 2024). Although these observations suggest that IGT events occur sporadically and independently among mycoheterotrophic orchids, the existence of plastome-derived sequences in the mitogenome of the photosynthetic relative *Phalaenopsis formosana* (Christenson) J.M.H.Shaw (Chen et al., 2020) indicates that such transfers are not exclusive to heterotrophic taxa. Consequently, it is difficult to characterize these IGT events as direct evolutionary adaptations driven by the transition to mycoheterotrophy.

Such transfer events are thought to be facilitated in part by tRNA-mediated recombination, which has been identified as a pivotal driver of IGT (Haberle et al., 2008; Sloan, 2013; Xiao et al., 2026). Among the eight tRNAs annotated in the mitogenome of *G. nudifolia* (**Figure 2**), *trn*N-GUU and *trn*R-ACG were located adjacent to plastome-derived regions homologous to *atp*A, *atp*F, and *ycf*1. In the typical photosynthetic orchid plastome, *ycf*1 is naturally adjacent to *trn*N-GUU and *trn*R-ACG, suggesting that the flanking region encompassing these two tRNAs and *ycf*1 was transferred as a single block into the mitogenome. In contrast, *atp*A and *atp*F were located far from these tRNAs in the plastome, implying that their presence near *trn*N-GUU and *trn*R-ACG in the mitogenome reflects independent transfer events. Sequence comparison of the two tRNAs between organelles further supports the occurrence of temporally distinct transfer events: *trn*R-ACG differs by only one base in the stem region, consistent with a relatively recent transfer, whereas *trn*N-GUU differs by seven bases, suggesting a more ancient event. One tetranucleotide SSR was identified near these tRNAs, and post-transfer rearrangements within the mitogenome cannot be entirely excluded as a source of sequence divergence. The colocalization of tRNAs with plastome-homologous regions and the differential divergence between the two tRNAs more parsimoniously support repeated, independent IGT events mediated by tRNA recombination.

Regardless of whether these plastome-derived regions originated from ancient transfers predating plastome gene loss, or from more recent events involving genes still present in the plastome, none of the plastome-homologous sequences identified in the mitogenome of *G. nudifolia* retain intact open reading frames, and they therefore show no evidence of retained coding capacity, persisting instead as nonfunctional remnants. Thus, while physical transfer of plastome sequences into the mitogenome has been extensive, functional gene transfer has not occurred. Comparable physical-functional dissociation has been documented across angiosperms (Wang et al., 2007), including mycoheterotrophic orchids (Kim et al., 2023b; Zhao et al., 2024; Kim et al., 2026). This distinction reinforces the view that despite their physical interaction through IGT, the two organellar genomes maintain functionally independent evolutionary trajectories.

### 4.4 Divergent selective regimes between the plastome and mitogenome under mycoheterotrophy

The structural and functional independence of the two organellar genomes described above raises the question of whether their evolutionary rates and selective pressures have also diverged in response to mycoheterotrophy. To address this, branch model analyses were applied to compare *ω* (*dN*/*dS*) between photosynthetic and mycoheterotrophic orchids for each organellar gene, with mycoheterotrophic lineages designated as the foreground branches (see Materials and Methods).

In the plastome, the loss of photosynthesis is associated with the progressive relaxation of purifying selection, potentially leading to the functional decay of genes that are no longer essential under a mycoheterotrophic lifestyle (Wicke et al., 2016; Graham et al., 2017). Our branch-model analyses revealed such relaxation in mycoheterotrophic plastomes, concentrated in loci associated with the photosynthetic apparatus and, most consistently, ATP synthesis, whereas Δ*ω* values for housekeeping genes such as ribosomal proteins and RNA polymerases remained near zero regardless of trophic mode (**Figures 5B, D**; **Supplementary Table 9**). Under the branch model, however, only *atp*I remained significant after correction for multiple testing, with several other ATP synthase genes marginally significant; we therefore refrain from characterizing this relaxation as genome-wide.

Because elevated *ω* under a branch model also cannot by itself distinguish relaxed from intensified selection, we further evaluated these loci using RELAX, which formally confirmed relaxation (*K* < 1) for several plastid ATP synthase genes (*atp*A, *atp*B, *atp*E, *atp*H, and *atp*I) as well as *psb*E and *mat*K (**Supplementary Table 10**). The concordance between the two approaches indicates that the elevated ω in these genes reflects a genuine relaxation of purifying selection rather than episodic positive selection, and that the signal, while concentrated in the ATP synthase complex rather than genome-wide, extends beyond the single gene significant under the branch model alone. Although the relaxation of selective constraints in heterotrophic plastomes has been documented previously (Barrett et al., 2014; Lam et al., 2018; Kim et al., 2019), the present results extend this paradigm by providing a direct comparison of the selective regime.

In contrast, the mitogenome showed no comparable, widespread shift in selective pressure. Branch-model analyses yielded uniformly low Δ*ω* values across all mitochondrial functional categories (**Figures 5A, C**), and RELAX identified significant shifts in only two mitochondrial genes—relaxation in *nad*6 and intensification in *atp*4—against a background of otherwise stable selective constraint. This contrasts with the plastome, where relaxation was concentrated in the photosynthesis-related and ATP synthase loci, and indicates that the mitogenome largely sustains purifying selection across trophic modes, consistent with the structural and functional stability documented in preceding sections. Previous studies have reported that the transition to heterotrophy is frequently associated with accelerated plastome substitution rates (Lam et al., 2018), whereas plant mitogenomes typically exhibit relative sequence stability despite their propensity for structural rearrangements (Palmer and Herbon, 1988). However, recent analyses of the fully mycoheterotrophic genus *Gastrodia* have revealed marked rate accelerations across both organellar compartments (Wang et al., 2025), suggesting that the degree of mitogenome rate shift may vary among lineages depending on the depth or duration of heterotrophic dependence.

A caveat concerns the difference in taxon sampling between the two organellar datasets (24 plastome vs. 30 mitogenome taxa), which arises from the limited availability of orchid mitogenomes. Because branch-model *ω* was estimated within each organellar dataset as a contrast between mycoheterotrophic and photosynthetic lineages, the direction of the selective shift in each compartment—relaxation in the plastome and stability in the mitogenome—is assessed relative to an internal photosynthetic reference and is therefore relatively robust to sampling differences. Nonetheless, the differing taxon composition means that the absolute magnitudes of Δ*ω* are not strictly comparable across compartments, and we accordingly interpret the plastome–mitogenome contrast as a qualitative difference in selective regime rather than a precise quantitative measure of divergence. A fully overlapping taxon set, which would allow direct magnitude comparison, is currently precluded by the scarcity of orchid mitogenome data and represents a goal for future work as more mitogenomes become available.

The maintenance of predicted editing sites across mycoheterotrophic mitogenomes is consistent with the retention of editing potential at the sequence level, but does not by itself demonstrate that post-transcriptional processing is preserved, as these sites were computationally predicted rather than validated by RNA-seq or cDNA data. We therefore interpret this pattern as exploratory rather than conclusive (**Supplementary Table 4**).

Taken together, the contrasting selective regimes of the two organellar compartments—relaxation in the plastome versus largely maintained constraint in the mitogenome—are consistent with differential functional dispensability, rather than the trophic transition alone, shaping their evolutionary trajectories in this lineage.

### 4.5 Phylogenetic relationships

Phylogenetic analyses based on mitogenome and plastome datasets yielded partially discordant topologies. In the mitogenome-based ML tree, *G. nudifolia* formed a clade with *C. lindleyana* within the Vanilloideae, which was recovered as sister to *Chamaegastrodia shikokiana* (Orchidoideae) (**Figure 4**). This phylogenetic position is likely attributable to two factors. First, Vanilloideae mitogenome data are currently restricted to two species (*C. lindleyana* and *G. nudifolia*), leaving the subfamily severely undersampled. This sparse sampling has also been a recurring limitation in previous plastome-based (Cameron, 2006; Cameron and Carmen Molina, 2006; Givnish et al., 2015; Kim et al., 2019; Pérez‐Escobar et al., 2021; Lee et al., 2025) and nuclear genome-based studies (Pérez‐Escobar et al., 2021; Pérez‐Escobar et al., 2024). Second, the long branch lengths exhibited by all four mycoheterotrophic genera (*Chamaegastrodia*, *Cyrtosia*, *Galeola*, and *Gastrodia*) suggest that elevated substitution rates may have contributed to the observed topology through systematic artifacts, such as long-branch attraction.

In the plastome-based ML tree, for which broader taxon sampling was available, the three sampled *Cyrtosia* species subtended *G. nudifolia* in a grade rather than forming a monophyletic group: *C. nana* was sister to *G. nudifolia* (BS = 100%), with *C. lindleyana* and then *C. septentrionalis* branching successively outside them (**Supplementary Figure 3**). Under this topology, *Cyrtosia* appears paraphyletic with respect to *G. nudifolia*, although the node placing *C. lindleyana* is weakly supported (BS = 49%) and sampling is limited, so this arrangement remains preliminary. This topology is consistent with previous plastome-based analyses, which showed that *Cyrtosia* and *Galeola* are closely related and form a sister clade to *Lecanorchis* (Givnish et al., 2016). Despite the long branches typically observed in mycoheterotrophic lineages, their phylogenetic relationships have been relatively well resolved in plastome-based studies (Lam et al., 2018). This suggests that the placement problem observed in our mitogenome tree primarily reflects the scarcity of Vanilloideae mitogenome data, rather than an inherent limitation of mitogenome markers.

These findings have two implications. First, our results tentatively suggest that the generic boundary between *Cyrtosia* and *Galeola* may warrant future re-examination. We emphasize, however, that this inference rests on limited organellar sampling (three *Cyrtosia* and one *Galeola* accession), weak support at the node underlying the paraphyly (BS = 49%), and an absence of nuclear evidence. Denser taxon sampling and independent nuclear markers are therefore required before any taxonomic revision can be justified. Second, taxon sampling density remains critical for the organellar phylogenomics of mycoheterotrophic lineages, where accelerated sequence evolution can exacerbate systematic artifacts. As additional mitogenome sequences become available across the Vanilloideae, the phylogenetic utility of mitogenome markers for resolving the relationships among mycoheterotrophic orchids will require reassessment.

## 5 Conclusion

We report the first paired plastome and mitogenome of *Galeola nudifolia*, a fully mycoheterotrophic Vanilloideae orchid. The transition to mycoheterotrophy is associated with contrasting selective regimes in the two organellar genomes: relaxation of purifying selection in the plastome, concentrated in photosynthesis- and ATP synthesis-related genes, whereas the mitogenome largely retains purifying selection, with only a few genes showing localized shifts. Although the physical transfer of plastome sequences into the mitogenome is extensive, none of the transferred regions retain intact open reading frames, indicating that the two organellar genomes maintain functionally independent evolutionary trajectories despite their physical interaction. Our plastome-based phylogeny recovers *Cyrtosia* as paraphyletic with respect to *G. nudifolia*; however, given the limited sampling and support, this arrangement is preliminary, and the generic boundary between *Cyrtosia* and *Galeola* will require re-evaluation with broader taxon sampling and nuclear data.

## Data Availability

The datasets presented in this study can be found in online repositories. The names of the repository/repositories and accession number(s) can be found below: https://www.ncbi.nlm.nih.gov/nuccore/PZ540802 and https://www.ncbi.nlm.nih.gov/nuccore/PZ593547-PZ593570.

## Supplementary material

Supplementary Figure 1 The photos of *Galeola nudifolia* Lour. **(A)** Habit, **(B)** Inflorescence, **(C)** Flower, **(D)** Seed pod, **(E)** close up inflorescence.

Supplementary Figure 2 The plastome map of *Galeola nudifolia*.

Supplementary Figure 3 Maximum likelihood phylogenetic tree constructed from 41 orchid species based on concatenated nucleotide alignments of 23 protein-coding genes and four rRNA genes of the plastome. The final alignment comprised 37,007 bp. Phylogenetic inference was conducted using IQ-TREE2 with best-fit substitution models (**Supplementary Table 3**). *Galeola nudifolia* and nonphotosynthetic, mycoheterotrophic species are highlighted in bold and red. Bootstrap support values are shown at the nodes, and asterisks (*) indicate nodes with 100% bootstrap support.

Supplementary Figure 4 The sequence divergence comparison between mitochondrial and plastid *trn*R-ACG and *trn*N-GUU.
